# Identification of Barriers for Active Disease Management and of Medication-Related Problems through Therapeutic Patient Education in Older Home-Care Patients

**DOI:** 10.3390/healthcare12020231

**Published:** 2024-01-17

**Authors:** Sabrina Grigolo, Barbara Bruschi, Diego Di Masi, Carla Bena, Lucrezia Greta Armando, Clara Cena

**Affiliations:** 1 Department of Philosophy and Education Sciences, University of Turin, Via Sant’Ottavio 20, 10124 Turin, Italy; sabrina.grigolo@unito.it (S.G.); barbara.bruschi@unito.it (B.B.); diego.dimasi@unito.it (D.D.M.); 2 Struttura Semplice Cure Domiciliari di Chivasso, Settimo Torinese, San Mauro Torinese, Via Po 11, 10034 Chivasso, Italy; 3 Department of Drug Science and Technology, University of Turin, Via Pietro Giuria 11, 10125 Turin, Italy; clara.cena@unito.it

**Keywords:** medication review, narrative interview, polypharmacy, home care service, potentially inappropriate prescriptions

## Abstract

Combining therapeutic patient education (TPE) with a medication review service could foster the adoption of appropriate lifestyles by patients and support care-providers in identifying strategies to improve the quality of prescribed care. This study aimed to identify barriers experienced by patients in managing their diseases and medication-related problems. This was a monocentric, case series, observational study involving home-care patients from the Local Health Authority ASL TO4. Patients were enrolled for a TPE intervention where drug therapies and patient habits were collected through narrative interviews. Medication review was performed to identify potentially inappropriate prescriptions (PIPs). Twenty patients (13 females) with a mean age of 74.7 years were enrolled. Patients had an average of 4.3 diseases and 80.0% of them were treated with ≥5 daily medications. The main PIPs involved ibuprofen, furosemide and pantoprazole. The qualitative analysis of the interviews identified seven macro-themes relating to different aspects of medication management: therapy; diseases; patient; patient journey; professionals; family and caregivers; drug information. The results of this study revealed some critical aspects related to the treatment path and healthcare professionals. These results will be used to plan educational interventions for polypharmacy patients to improve medication adherence and the understanding and management of diseases.

## 1. Introduction

Individuals diagnosed with a chronic condition need to modify their lifestyle in order to manage their new health condition, which often requires external interventions by different professionals to understand what actions need to be put in place to ensure quality of life [1,2]. In addition to the intervention of healthcare professionals such as general practitioners (GPs) and pharmacists, who are responsible for prescribing and counselling on the necessary medications to treat the new condition, another aspect that should not be underestimated in disease management is the implementation of a therapeutic patient education (TPE) intervention. TPE has been defined by the World Health Organization (WHO) as *helping patients to acquire or maintain the competencies they need to manage their lives with a chronic disease as well as possible* [3]: for chronic patients, particularly those with comorbidities, learning skills and healthy behaviours is fundamental to maintaining an adequate quality of life. In fact, acquiring such behaviors can not only delay complications arising from the disease, but also promote self-management, enabling the patient to integrate his or her disability into daily life [4,5,6,7,8,9]. In recent years, several studies [10,11] have shown that educational interventions can improve disease outcomes for various chronic conditions and populations. This contributed to the recognition of TPE as a basic and lasting component of therapeutic management by several stakeholders worldwide, who have promoted its integration into international guidelines for the prevention and treatment of chronic diseases [11,12].

Educational research in healthcare starts from the identification of the individual’s necessities, which can prompt a naïve patient to acquire new skills to manage his or her own health in a familiar living environment [2]. The context in which the patient lives, their degree of acceptance of the disease, their abilities and their level of autonomy are some of the factors that influence patients’ motivation and learning attitude [4]. Therefore, an interdisciplinary approach is the best option to ensure the patient is actively engaged in the management of the disease. Education, sciences, medicine, nursing, drug science and anthropology are just some of the disciplines that come into play in educational research in the healthcare sector. As shown by several studies [13,14,15,16,17,18], patient engagement has a positive impact on healthcare processes and health; moreover, patients who are more actively engaged in their care are generally more adherent to their physician’s recommendations [19] and drug prescriptions [20].

The aim of this observational study was to identify the main barriers and elements influencing the management of diseases from the perspective of patient empowerment and patient-centred care. The secondary objective was to perform a medication review to identify potentially inappropriate prescriptions (PIPs) and other medication-related problems in the patients’ therapies.

Several studies [21,22,23,24] tested narrative approaches as tools to understand patients’ beliefs in order to improve disease management. These interventions are particularly important for patients with chronic diseases requiring polypharmacy, a condition which increases the risk of medication-related problems and the difficulty of managing complex regimens. Another population that could benefit from TPE interventions is older adults, who, in addition to experiencing a progressive decline in their physical and cognitive abilities, often experience increased medication-related problems (e.g., due to the prescription of inappropriate medications, overly complex medication regimens, the occurrence of adverse reactions or poor adherence to medications) [25]. Although healthcare professionals may advise on the correct therapeutic behaviour, it is ultimately up to the patient to decide whether or not to take prescribed medications, and when and whether to modify or supplement their pharmacological treatment. Combining a TPE intervention aiming to understand how patients manage their diseases with a medication review service could not only foster the adoption of an appropriate lifestyle by patients, but also support care providers in identifying strategies to improve the quality and safety of prescribed care.

## 2. Materials and Methods

### 2.1. Study Design

This monocentric observational study received a favourable opinion from the Ethical Committee of the healthcare units of competence; the study was launched together with a broader national project called *Sistema Integrato di Lifelong Learning per la Valorizzazione del capitale umano, l’Educazione alla cittadinanza attiva e il Recupero delle capacità cognitive* (SILVER). Patients were randomly selected by healthcare professionals in the home-care service of the south-east area of the ASL TO4 (Piedmont, Italy), and they were enrolled from September 2022 to December 2022. The home-care service includes medical, nursing, rehabilitation and care services provided at home to citizens with varying degrees of socio-medical difficulties that hinder access to healthcare services. This service includes a home visit by a nurse once a week or every two weeks, as well as medical or physiotherapy services as required, and it involved 130 older adults in 2022. The ASL TO4 is a local health authority comprising 5 districts, or areas, in the north-west of the city of Turin, with approximately 520,000 inhabitants.

The study was conducted in full accordance with the General Data Protection Regulation (EU) 2016/679.

### 2.2. Participants

Participants deemed eligible and willing to participate in the study were referred to the principal investigator (PI). The inclusion criteria were as follows: patients over 60 years in home care in the most populated area of the ASL TO4, with 3 or more medications per day and at least 2 chronic diseases, who signed their informed consent. To be enrolled in the study, patients also had to have a caregiver present during the interview. Individuals using the home-care service are self-sufficient patients with varying degrees of disability related to the presence of one or more chronic diseases, such as malignancies, cardio- or cerebrovascular diseases, rheumatological diseases or dysmetabolism. Narrative interviews were conducted with all patients who met the inclusion criteria, while the following exclusion criteria were applied for the medication review: incomplete data on pharmacological treatment and need of palliative care.

The PI explained the study design and collected informed consent in order to conduct narrative interviews with the enrolled patients. Narrative interviews were the instrument through which data on pharmacological therapy and other information related to the story with the disease and its management were collected. Only the PI was authorized to know the identity of the participants, as narrative interviews were transcribed in anonymised form prior to transmission to the other investigators.

### 2.3. Data Collection

All the analysed data were collected through narrative interviews. Narrative approaches have been recognized as effective strategies to promote patient-centred healthcare pathways and patient empowerment, defined as *a process through which people gain greater control over decisions and actions affecting their health* by the WHO [26]. Narrative interviews were performed by SG, the PI of this study with expertise in educational processes, clinical research methodologies and project management. They were performed in the homes of the enrolled patients in the presence of the medical staff of the home-care service and the patient caregiver. They consisted of unstructured interviews without standardised questions and responses, and with the questions administered in an unpredictable order. The questions administered to the patients were open questions and mainly concerned three areas of therapeutic education: the story of the disease, the list of prescribed medications and the patient’s approach to their own condition. Notably, narrative interviews allow for patients to talk about their “journey” with the disease and, at the same time, help the healthcare professional to better understand patients’ needs, resources and perspectives, creating positive effects on care outcomes [27]. Narrative interviews comprised four moments:

1. Introduction to the study and explanation of the research methodology;
2. Storytelling, in which the interviewee is invited to talk about their disease with the following question: “What aspect would you like to start from when talking about your experience with drug therapy management?”;
3. Possible questions to fill any gaps and to ask for clarification, such as “What strategies or activities have you put in place to implement the therapy correctly?” and “Can you list some activities you do before or after taking the therapy?”;
4. Explanation of the study phases following the interview.

During the interview, the investigator placed the interviewee at the centre of the process using non-verbal encouragement and simple language, without interrupting the storytelling.

All interviews were audio-recorded and then manually transcribed into anonymous files for the analysis by SG. Each interview lasted approximately 45 min; each transcription was verified by one of the authors listening to the recording. The information on the pharmacological therapies (e.g., medication name, dosage and time of intake) prescribed to patients was obtained from the interviews and was used by researchers with expertise in the field of medication review and prescriptive appropriateness to prepare the therapy records of the enrolled patients. For each patient, a Summary Therapeutic Sheet (STS) was prepared (Supplementary Materials, Figure S1), including:

- Anonymous unique patient identifier;
- Patient’s date of birth;
- Patient gender;
- Patient age at the time of the interview;
- Diagnosis;
- Daily drug therapy, including the name and dosage of each medicinal product and of the active ingredient, the condition for which it is used, the pharmaceutical form, the daily dosage, whether to take it with meals, the times of intake, what to do if a dose is missed and precautions for use;
- Weekly, monthly/periodic or as needed therapy;
- Any notes from the doctor, patient/caregiver or pharmacist.

In case of doubts or inconsistencies, the medical staff of the home-care service was contacted by the PI to confirm or modify the therapy.

The collected data were analysed according to the specific aim of the study: a qualitative analysis of the narrative interviews to identify barriers and elements influencing the management of the disease and a description of medication-related problems in patients’ therapies.

### 2.4. Qualitative Analysis of Narrative Interviews

NVivo 12 software (https://lumivero.com/products/nvivo/, accessed on 1 May 2023) was used to import transcriptions and to code them in order to derive recurring categories (or themes) and macro-categories (or macro-themes). The qualitative analysis of the interviews was carefully performed by two authors (DDM and SG) with expertise in qualitative data analysis, who read the interviews and discussed emerging themes and macro-themes. The Grounded Theory method [28] was applied to code patient inputs: a 3-level coding was used to derive concepts (the patient’s words), categories (coarse grouping of concepts) and macro-categories (groupings of categories to identify recurring macro-themes) from the interviews and their frequencies were measured. In brief, the Grounded Theory consists of patients describing their lived experiences through the administration of narrative interviews in order to understand a particular phenomenon, adopting three encodings: the basic concept as referred to by the interviewee (units meaning), the first classification of basic concepts (units of analysis) and the classification of units of analysis to identify third-level codes (macro-themes). It should be emphasised that units with the same meaning can have different interpretations that will produce different second-level encodings. These operations are carried out by experts in the field, who must agree on the selection of themes and macro-themes.

### 2.5. Tools for Medication Review

The pharmacological therapies that were collected were analysed to identify medication-related problems, including PIPs. LGA and CC independently identified medication-related problems for each patient, which were then compared and discussed to prepare the final therapy review report. Reports included suggestions on how to improve pharmacological therapy and reduce PIPs; the patient’s name was replaced by an anonymous identification code in all reports. Reports were sent to the medical staff of the home-care service, together with the STSs, to enable the optimisation of the prescribed therapies, supporting discussion with the patient/caregiver regarding problems with the current therapy and suggesting changes to the therapies.

Medication-related problems were assessed in terms of drug–drug interactions or DDIs (contraindicated and major according to the Micromedex^®^ database) [29], Beers criteria (2019 update) [30], Screening Tool of Older Persons’ Prescriptions (STOPP) version 2 [31] and anticholinergic cognitive burden (ACB) [32]. The Beers and STOPP criteria are internationally recognized tools consisting of validated lists of PIPs in older adults; they also include additional information, such as the severity of the recommendation and clinical advice to avoid medication-related problems. Microsoft Excel spreadsheets were prepared to identify medication-related problems from STSs and to classify PIPs into three categories: PIPs that are not applicable to the specific patient; conditional PIPs, i.e., medications that are potentially inappropriate in older adults with specific conditions that could not be extrapolated from the interviews or from the discussion with the physician; confirmed PIPs, i.e., medications that are potentially inappropriate or that should be used with caution in most older adults. PIPs that were not applicable were not considered for the medication review.

### 2.6. Data Analysis

The population was analysed by describing the characteristics of the enrolled patients. A review of the medications and suggestions regarding the optimisation of therapy were elaborated by researchers specializing in drug utilisation research using validated tools (see Section 2.5). All data were analysed using the Microsoft Excel software 2019 (version 2312) between February 2023 and July 2023.

## 3. Results

### 3.1. Characteristics of the Population

The medical staff of the home-care service identified 40 patients that were eligible for the study. All patients underwent an evaluation of their clinical condition by the medical staff of the home-care service in order to be admitted to the service (clinical data not available). Almost half of the eligible patients refused to participate because they did not want to welcome the investigators into their homes. A total of 23 patients (14 females) signed the informed consent and agreed to participate in the study. After conducting the narrative interviews, three patients were excluded due to incomplete therapy data (two patients) and need for palliative care (one patient). A medication review was carried out for the remaining 20 patients (13 females); the general characteristics of the study population are summarized in Table 1. The majority of patients (13 out of 20) had a situation of major polypharmacy (5–9 prescribed daily drugs) according to the classification of Masnoon et al. [33]; four patients had minor polypharmacy (2–4 prescribed daily drugs), while three patients had excessive polypharmacy (10 or more prescribed daily drugs). To count daily and periodic drugs, the different active ingredients taken by the patient were considered, so medications containing fixed combinations of two active ingredients were counted as two drugs; the number of dosage units per day was considered as the number of times per day the patient had to take a drug, including periodic administrations. The number of daily administrations ranged from 4 to 22: 10 patients took from 5 to 9 dosage units per day, 8 patients took more than 10 and 2 patients took 4 dosage units per day.

The most prescribed drug in the study population was insulin (10 patients), aspirin (7 patients), pantoprazole (7 patients), atorvastatin (6 patients), furosemide (6 patients), bisoprolol (5 patients), metformin (5 patients) and paracetamol as needed (4 patients). The pathologies that were found were rather heterogeneous: among the most frequent were diabetes mellitus (12 patients), cardiac diseases (11 patients), arthrosis (10 patients), hypertension (9 patients), metabolic disorders (4 patients) and malignancies (4 patients). Table 2 shows the list of diseases reported directly by each patient or deduced by the authors from the medications taken.

### 3.2. Narrative Interviews: Qualitative Analysis

A total of 23 interviews were analysed through NVivo, corresponding to the 23 patients (14 females) initially enrolled in the study. The three patients excluded from the medication review were included in the analysis of the interviews, with the aim of identifying barriers to disease management. The average duration of each interview was 45 min. Interviews were conducted in Italian and the results were translated into English by the authors for dissemination. Seven macro-themes were identified by grouping forty-three themes extrapolated from the interviews:

1. Therapy;
2. Diseases;
3. Patient;
4. Patient journey;
5. Professionals;
6. Family and caregivers;
7. Drug information.

The complete list of themes and macro-themes, along with their frequencies, is shown in Table 3, which also includes example quotes from patients and their identification number (P1, P2 etc.).

**Figure 1:**
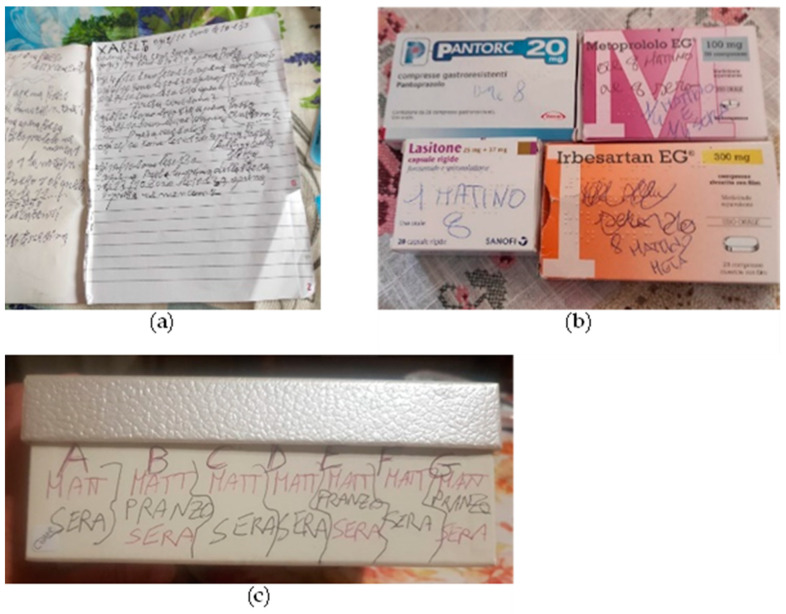
Strategies adopted by patients and/or caregivers to remember the correct therapy to take: (**a**) diary with the daily therapy written day by day; (**b**) medication boxes with the daily dosage written by the patient; (**c**) cardboard box containing the daily medication with the dosage written on it.

From the analysis of the interviews, it was possible to identify the main barriers to informed disease management. Most patients (13 out of 23) reported difficulties in managing the intake of the pharmacological therapy, mainly due to the lack of a GP-verified summary document with the daily dosage. Figure 1 shows some of the strategies adopted by patients and caregivers to avoid medication errors: notebooks with the daily therapy written for each day (panel a); medication packages with the daily dosage written on them (panel b); daily medication boxes prepared every morning.

Almost all patients (22 out of 23) complained of therapies being too complex (injectable medications) or there being too many daily medications. Twelve out of twenty-three patients admitted to modifying their prescribed therapy without consulting their GP due to the presence of side effects, while others (twelve out of twenty-three) stated that they did not perceive the benefits of the prescribed therapy and instead associated it with a worsening of their quality of life (e.g., onset of weakness, nausea, confusion).

#### Macro-Themes: Examples of Patients’ Experiences

Among the most recurring themes and macro-themes extracted from a qualitative analysis of the interviews, a number of aspects emerge that are worth mentioning. The macro-theme “Therapy” represents the most relevant aspect, with almost all patients talking about this in their story of the disease. “Therapy management”, “drug therapy” and “therapy intake” are the most frequent elements of this macro-theme; they refer, respectively, to knowing how to manage the therapy, including the procurement, conservation, preparation and disposal of drugs, as well as to the actual intake of the drug and to the different types of drugs and routes of administration. The majority of patients perceive that they take too many medications, have difficulty preparing them or have difficulty scheduling their intake throughout the day.

*“There’s the Voltaren* (author’s note: diclofenac gel) *that I put on my hands every night because I get crazy pains at night. Then I have Tachipirina* (author’s note: acetaminophen), *but I can’t even take that much because I have kidney problems. Now I’ve had an itch in my back for a few days. They gave me this when they operated on my foot. I take two a day. But I’m afraid it is harmful now because for a week I’ve had an itch in my back, just in my back. Then there are other anti-inflammatories that I take every day”.*

(Patient 14)

The methods adopted by patients and caregivers to remember when and which medication to take are variable, but the most common is the act of writing and rewriting the therapy every day on sheets of paper, preparing daily or weekly boxes containing the complete therapy or writing intake information on the medication package.

*“I write the therapy in my diary every day”.*

(Patient 10)

Other patients separate the medications in different places in the house or store them in different bags or containers according to the time of intake:

*“In order not to make mistakes I do this* (author’s note: the interviewee points to several bags containing medications)*: these are for the morning, these are after noon*”.

(Patient 11)

Polypharmacy management also assumes the patient’s ability to self-manage medications, not only during their preparation and intake but also by recognising possible complications at an early stage.

“*I don’t take a lot of drugs now because I have some problems. Now I’m doing a test, it’s the third day I’m taking Aliflus* (author’s note: salmeterol + fluticasone aerosol), *if it also gives me problems we’ll see. I can already hear my voice getting a bit hoarse*”.

(Patient 18)

A continuous adaptation of the treatment scheme, whether independently or not, and requests for changes in drug therapies represent other two recurrent elements in patients’ medical stories.

*“I have tried some drops, some granules, but I can’t keep them in my stomach. So, I pointed this out to the doctor and now I do some therapies intravenously or I take tablets if they are available”.*

(Patient 8)

The second most frequent macro-theme is “Diseases”, with “disease effects” and “disease complications” as recurring themes. For the interviewed patients, the disease is the reason they take the therapy and the starting point for talking about their condition. These elements bring out a twofold aspect of the patients’ perception of the quality of care: in some cases, how negative their perception of care is becomes clear:

*“I’ve had* this *severe asthmatic condition, respiratory insufficiency, for 33 years. Taking 50 mg a day of cortisone has been killing me. In those years there was only that life saver. It caused me osteoporosis”.*

(Patient 19)

In other cases, their perception of the quality of care is quite positive:

*“Colon* cancer. *15 cm […] If I didn’t undergo surgery within an hour, I was dead. They opened me up, pulled out a long part of my intestine, after which they left me open for 3 days attached to a machine. Then they fixed it and it all went well. They put the ileostomy in and I’ve had it ever since. I honestly don’t have a problem with it”.*

(Patient 9)

The third aspect to consider is “Patient”, understood as the set of strategies and reactions of the patient and their reconciliation of the therapy and its effects on daily life. Increasingly, patients want to actively contribute to their own care by agreeing with their doctor regarding the most effective and correct strategies for themselves. The strategies implemented by patients or caregivers may derive from their personal experiences or beliefs:

*“They* wanted *to amputate because the leg no longer worked. […] I took liquid betadine* (author’s note: povidone iodine solution). *I’d soak the foot in it and then add mimosa soap and hydrogen peroxide, a little penicillin. I’d make a mixture, I mix it and soak the foot a quarter of an hour. Then I’d take it all out and throw it away”.*

(Patient 4)

Patients’ reactions to therapies and diseases may vary and depend on the individual situation: in some cases, patient engagement prevails, demonstrating the patient’s ability to self-manage the recommendations they receive. In other cases, denial and/or forgetfulness of the patient’s condition prevails.

*“I do the* checks *the doctor tells me. So far, I have never had any problems”.*

(Patient 1)

*“Several years ago, I used to pee a little red. Two, three days and then it was white for a few days. I did nothing, but now I know I should have done something immediately”.*

(Patient 5)

Reconciling the therapy and its effects on daily life represent two different but closely related areas. In fact, taking medication does not only imply the that the patient is able to identify any effects that need to be managed (e.g., taking the diuretic in the morning before going on a trip), but also they can manage their intake of different pharmaceutical forms and different routes of administration.

*“You can delay by half an hour and this can happen on Sundays when we eat later, otherwise I always try to be punctual and precise”.*

(Patient 6)

The fourth macro-theme that was analysed is the “Patient journey”, i.e., the set of check-ups and exams that the patient undergoes to monitor the progress of the disease. Half of the interviewees underlined their difficulty in managing periodic checks on their own, as well as reiterating the stress caused by the long period of illness.

*“6, 7 years ago I fell at home and they operated on me. I spent a month in the hospital, two months at home and three months in a long-term care facility to do rehabilitation”.*

(Patient 10)

Another aspect that emerged was a lack of communication among healthcare professionals and with the patient:

*“A nurse who specialized in ostomies came to my house that time when the ostomy couldn’t stay attached. If you refer problems with ostomies in the hospital, they don’t listen to you. On the contrary, she is very attentive to the patient’s quality of life. I walk, I do a certain type of thing, I need a certain type of ostomy. Someone who is bedridden, very ill, needs another ostomy. Instead, specialists in hospitals all give you the same ostomy”.*

(Patient 7)

The fifth macro-theme, “Professionals”, is related to the healthcare professionals involved in patient care and to hospital networks. In most cases, the patients that were interviewed stated that the relationship with their physician was good, with the exception of the excessive waiting times for specialist visits and prescription medication, which may result in a loss of motivation.

“*We waited for 2 years* (author’s note: for the visit with the surgeon)*”.*

(Patient 9)

The hospital network consists of the referring healthcare facilities and the physicians, nurses and pharmacists involved in the care pathways. In particular, some patients mentioned their relationship with the community pharmacist, which is based on mutual knowledge and trust. This plays a key role in pharmaceutical care, as it provides a solid basis for collaboration between healthcare professionals and for an effective and personalized management of drug treatment.

*“There is a relationship of trust with the pharmacist who already knows that I take certain medicines. When I go without a prescription, they give me the medicine anyway. I come back the next day and hand in the prescription”.*

(Patient 1)

Another aspect that emerged from the analysis of the interviews is the need, for the majority of patients, to have support from a caregiver or family member in their management of the disease (macro-theme “Family and caregiver”). If this support is lacking, the patient may experience social isolation, which may affect the person’s ability to manage the disease properly.

*“He did everything. I’m in the hands of my son. He takes care of everything”.*

(Patient 5)

### 3.3. Medication Review

Therapy data were complete for 20 patients, and were analysed to describe medication-related problems and PIPs. All patients had at least one medication-related problem according to the Beers criteria, STOPP criteria and DDIs. Specifically, medication-related problems ranged from 0 to 11 for the Beers, from 0 to 9 for the STOPP and from 0 to 15 for major DDIs (Supplementary Materials, Table S1).

**Figure 2:**
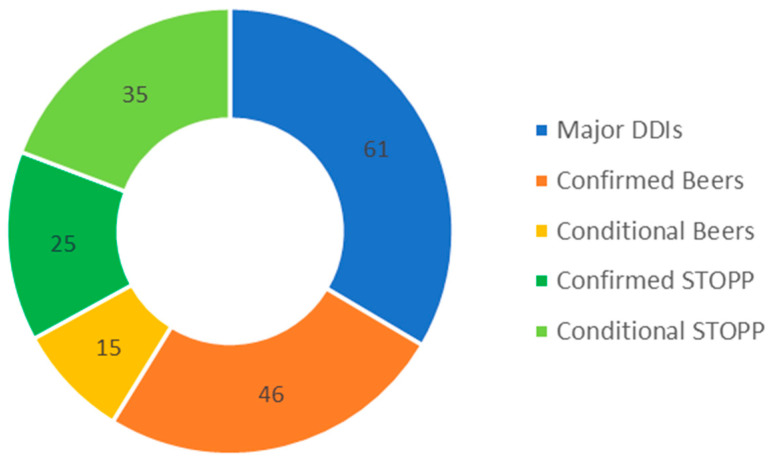
Medication-related problems identified in the prescriptions of the study population. Numbers represent the number of medication-related problems for each category.

A total of 182 medication-related problems were identified in the therapies of the study population, which were classified into five categories, as shown in Figure 2.

Sixteen patients had at least one PIP according to the Beers criteria, ranging from 1 to 11 different PIPs for the same individual. A total of 61 PIPs, according to the Beers criteria, were identified in the prescriptions of the study population: 46 confirmed PIPs and 15 conditional PIPs. The drugs most frequently associated with both confirmed and conditional PIPs, according to the Beers criteria, were pantoprazole (seven PIPs), furosemide (six PIPs), ibuprofen (five PIPs) and alprazolam, rivaroxaban and sertraline, with each corresponding to four PIPs. The most frequent condition that could not be verified to confirm the actual presence of conditional PIPs was a history of falls or fractures (six PIPs).

**Figure 3:**
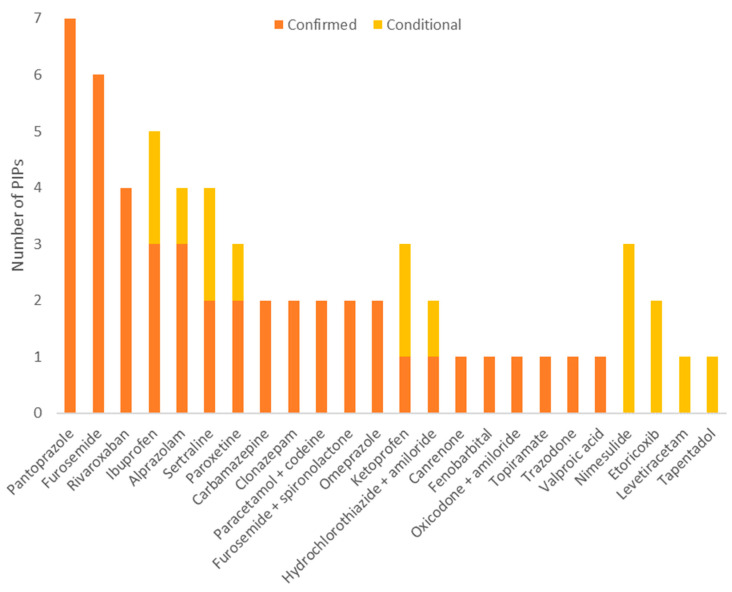
PIPs according to the Beers criteria considered for the analysis.

**Figure 4:**
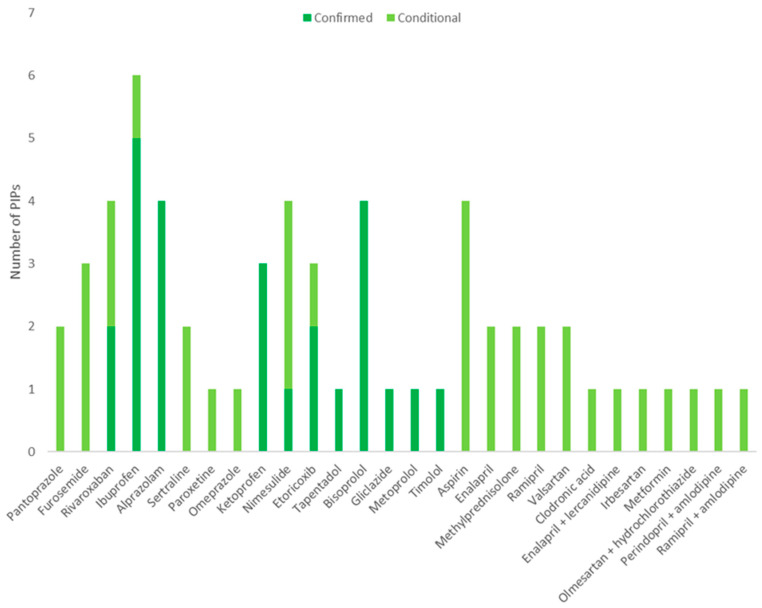
PIPs according to the STOPP criteria considered for the analysis.

The total number of PIPs according to the STOPP criteria was 60: 25 confirmed PIPs and 35 conditional PIPs. Only 1 patient out of 20 had no PIPs according to the STOPP criteria, while 19 patients had at least one PIP, ranging from 1 to 9 different PIPs. The most frequent PIPs according to the STOPP criteria involved ibuprofen (six PIPs) and alprazolam, aspirin, bisoprolol, nimesulide and rivaroxaban, which counted for four PIPs each. Conditional PIPs according to the STOPP criteria most frequently concerned the presence of hyperkalaemia (11 PIPs) and of previous peptic ulcers or gastrointestinal bleeding (9 PIPs). Figure 3 and Figure 4 show, in detail, the PIPs considered for a medication review for both the Beers criteria (Figure 2) and the STOPP criteria (Figure 3).

It should be noted that a drug can count for more than one PIP for the same individual according to the Beers and the STOPP criteria: e.g., paroxetine (an antidepressant of the selective serotonin reuptake inhibitor class) has three different PIPs according to the Beers criteria and one according to the STOPP criteria, as shown in Table 4.

The Micromedex^®^ database was consulted on March 2023. No contraindicated DDIs were found in the study population, while 61 major DDIs were found: 7 patients out of 20 did not have major DDIs, 9 patients had 1–5 DDIs, and 3 patients had 8, 11 and 15 DDIs, respectively. Only the DDIs of aspirin—metformin (5 DDIs out of 61), aspirin—furosemide (3 DDIs out of 61) and aspirin—etoricoxib (2 DDIs out of 61) were common to several patients, while the remaining 44 had only one occurrence, underlining the heterogeneity of the study population. After grouping the active ingredients by pharmacological class, 25 unique DDIs were obtained, as shown in Table 5.

Finally, the ACB score was calculated to assess the risk of anticholinergic effects in the prescribed therapies: 6 patients out of 20 were not taking anticholinergic drugs; 6 patients had an ACB score of 1; 5 patients had a total ACB score of 2; 3 patients had a score of 3, 5 and 6, respectively. ACB score ranged from 0 to 6, with no differences between males and females. Table 6 shows the drugs associated with anticholinergic effects and the number of patients on the drug.

A total of 20 STSs containing the pharmacological therapy were collected from the narrative interviews, then prepared and sent to the medical staff of the home-care service, together with 20 short reports including the PIPs that were identified and suggestions on how to reduce PIPs. In addition to highlighting possible therapy-related risks, a total of 29 suggestions on how to optimise drug therapy were made and are summarised in Table 7.

## 4. Discussion

This study aimed to identify the main barriers to proper disease management through a TPE intervention including an evaluation of appropriateness of prescriptions for the enrolled patients. To the best of our knowledge, this is the first study combining an educational intervention with a quantitative analysis of prescriptions in a population of chronic patients. Previous studies [22,23,24,25,26,27] using narrative approaches have focused only on the educational part to explore patients’ motivators and perception towards a more active disease management.

Due to the nature of the home-care service, the population enrolled in this study was quite heterogenous, in terms of both the number and type of drugs used, as well as comorbidities and general health status. The collection of data on the pharmacological therapies taken by patients was challenging because some patients did not know how to take their medications correctly or the reasons for their use, leading to changes in treatment that they did not discuss with the medical staff of the home-care service. For this reason, patients and caregivers particularly appreciated the opportunity to tell their disease story and to be listened regarding the difficulties encountered in the management of polypharmacy.

The qualitative analysis of interviews showed that medication management and living with the disease are complex operations, driven by the patient’s beliefs and perception of the disease. As observed by Fadare et al. [22], patients adopt various tools and strategies to integrate polypharmacy into their daily life. These strategies may involve the identification of specific places within the home in which to store certain medications, the adoption of routines to associate medication intake with specific activities or the self-adjustment of the dosing schedule and, in some cases, the decision of whether or not to take a drug without consulting the GP. The critical aspects that are most worth mentioning are related to the macro-themes “Patient journey” and “Therapy”. Some patients reported problems in communication between healthcare professionals, who may have conflicting opinions and/or are not updated on patients’ ongoing condition. Moreover, the different tools used by patients to correctly take their therapy indicate a lack of a summary information about the prescribed therapy: patients reported receiving information from the physician during visits on how to take their medications, but often did not remember this when they returned home because it was not written down. Related to this, another common undesirable situation among older patients is the consumption of the “stock of medications kept at home” until it is exhausted, because the change in their therapy has not been understood. This was evidenced by the various cabinets and trunks containing numerous different medications observed in patients’ homes. Similar findings were found by Midena et al. [21] on patients with macular degeneration and by Picchi et al. [27] on diabetic patients. A macro-theme that was poorly represented, contrary to expectations, was “Drug information”. There may be several reasons for this under-representation, including prior knowledge, which leads to the need for information already being met. As Tuckett et al. states [34], chronic patients who have been in treatment for many years are patients who are aware and knowledgeable about their own health status and are able to identify the necessary information to manage their own health. Other reasons are related personal factors of individual patients (e.g., motivation, perspectives, expectations, level of activation).

Patients included in the analysis of medication-related problems had an average of 4.3 chronic diseases; this condition of comorbidity could significantly affect the quality of life of older patients, impacting their general health, functional abilities and daily independence. The average number of different daily active ingredients was 7.4, highlighting the fact that most patients (16 out of 20) had major or excessive polypharmacy. Although the degree of complexity among the enrolled patients is not known (it is beyond the scope of this study), it can be assumed that they present varying degrees of complexity related to the presence of comorbidities and polypharmacy, in line with the fact that they were accepted by the home-care service. As many studies have shown [35,36,37,38,39], the results of the medication review confirmed the presence of medication-related problems and PIPs in the therapies of older chronic patients, highlighting the importance of periodic re-evaluations of prescribed medications or of a medication review during transitions of care to improve the concordance between patient and GP. The most frequently identified PIPs according to both the Beers and the STOPP criteria were ibuprofen, furosemide, pantoprazole and rivaroxaban. These are preventable PIPs that are already included in validated recommendations and guidelines for prescribing medications in older patients, which, although they do not have a high risk of causing serious adverse reactions, could cause side effects and make the therapy more complex and difficult to manage. These results are consistent with other studies that included an evaluation of the prescriptive appropriateness of chronic pharmacological therapies [36,37]. This underlines the importance of involving a drug expert, such as a pharmacist or a researcher on drug utilization, in the patient care pathway: although some of the evidence [40,41,42,43,44] associated the presence of a drug expert with positive health outcomes, this figure is still not systematically involved in clinical practice at a national level.

### Strengths and Limitations of the Study

This study presents several limitations. First, a limited number of patients were enrolled. This was partly due to the coronavirus disease 2019 (COVID-19) pandemic that greatly delayed the start of the project. Because of the COVID-19 pandemic, home-care patients were reluctant to welcome the interviewer and the home-care staff into their homes. Other major limitations of the study related to the use of patients as the sole source of data collection and the lack of feedback from the physician in the home-care service on reports of medication-related problems and PIPs. Future interventions should also include a return to patients treated with an STS prepared by the physician and the drug expert, which could help patients better manage their polypharmacy. A collaboration between the medical staff in the home-care service and researchers would also be helpful in gaining a better understanding of the clinical conditions of the enrolled patients, which, among other things, could allow for an assessment of patients’ complexity.

The main strength of the study is the novelty of the approach that was taken, which combines education, patient narrative and a medication review. Conducting the interview at the patient’s home also made it possible to obtain more transparent and honest information, even from the most sceptical patients, as they felt safe in a familiar place like their own home.

## 5. Conclusions

The approach tested in this study, particularly the adoption of narrative interviews conducted by healthcare professionals with patients and caregivers as the survey method, proved to be fruitful as a tool aiming to stimulate patients’ ability/need to continuously rethink their health condition in order to improve it [45]. The results confirm the importance of the concordance, or therapeutic alliance, between the patient and GP, which is one of the most relevant elements in achieving improvements in the management of one’s own health condition, as defined by De las Cuevas et al. [46]: in concordance with this is *the extent to which patients are successfully supported both in decision-making partnerships regarding their medicine and in taking their medicine*. The recognition of medication-related problems in the therapies of home-care patients enabled the identification of areas of improvement to optimize prescribed care. TPE represents an important instrument, both for obtaining standard information about the disease and for learning about patient’s emotions regarding the disease.

The results of this study will be used to plan educational interventions for older patients with comorbidities and polypharmacy to improve medication adherence and the understanding and management of patients’ diseases.

## Figures and Tables

**Table 1:** General characteristics of the study population.

	Total	Males	Females
Study population, n	20	7	13
Age at the time of the interview, range	61–96	61–84	64–96
Number of daily drugs, range	3–15	4–15	3–15
Number of weekly/monthly/cyclic drugs, range	0–5	0–5	0–3
Number of drugs as needed, range	0–6	0–1	0–6
Number of dosage units per day, range	4–22	6–22	4–16
Number of chronic diseases, range	2–6	2–6	3–6

**Table 2:** Diseases affecting the study population.

Patient Identification Number	Diseases Reported Directly by Patients or Deduced from the Medications Taken
P1	Type 1 diabetes Heart disease with previous myocardial infarction Hypertension Chronic kidney disease
P2	Type 2 diabetes Hypertension Major depressive disorder Anxiety Dyslipidemia
P3	Epilepsy Osteoporosis Polyarthrosis Hypertension Dysmetabolism and obesity
P4	Type 1 diabetes Hypertensive cardiopathy
P5	Bladder cancer Polycythemia vera Hypertension
P6	Hypertensive cardiopathy Type 2 diabetes Polyarthrosis
P7	Dysmetabolism Rectal cancer
P8	Chronic senile deficiency Hyperuricemia Anemia Hypertension Diverticulitis
P9	Lower-limb thrombosis Hypertensive cardiopathy Hypothyroidism Arthrosis with previous hip fracture
P10	Valvular heart disease Type 1 diabetes Peripheral vascular disease Asthma Arthrosis Dysmetabolism
P11	Type 1 diabetes Hypertensive ischemic heart disease Chronic kidney disease Polyarthrosis Anemia Gout
P12	Hypothyroidism Type 2 diabetes COPD Arthrosis Gout
P13	COPD Spinal column collapses Arthrosis Mitral valve disease Type 2 diabetes Breast cancer
P14	Hypertension Epilepsy Polyarthrosis with osteoporosis
P15	Hypothyroidism Hypertension Arthrosis
P16	Asthma Heart disease Hypertension Glaucoma
P17	Pneumonia COPD Asthma Type 2 diabetes
P18	Urethral disease Heart disease Type 2 diabetes Gout Hypercholesteremia Arthrosis with osteoporosis
P19	Type 2 diabetes Parkinsonism Major depressive disorder Heart disease Hypertension Benign prostatic hyperplasia
P20	Type 2 diabetes Heart disease Dysmetabolism Hypercholesterolemia Hyperthyroidism

Abbreviation: P, patient; COPD, chronic obstructive pulmonary disease.

**Table 3:** Themes and macro-themes extrapolated from narrative interviews. The number of patients who expressed a certain concept (Pt.—patients) and the number of times the concept was repeated in all interviews (Oc.—occurrences) are also reported.

Example Patient Quotes (1st Level Coding)	Themes (2nd Level Coding)	Macro-Themes (3rd Level Coding)	Pt. (n)	Oc. (n)
“I take medications from 8 a.m., every 2 or 3 h. I have medication to take until after dinner before going to sleep”. (P1) “I don’t have a sheet with the medication schedule. The ones I take in the morning I put over there. The ones I take in the evening I put over here. These are the thyroid ones, 100 (*AN*, *mg*) alternating with 75”. (P13) “I was sick and I realised that it was the cause (*AN*, *fluticasone + vilanterol*). I suspended it and the next day I was reborn. I even went for a 10 km walk in Genoa”. (P18) “By taking the non-original medications I was more sick than well”. (P6)		**Therapy**	**22**	**253**
	*Therapy management*		22	82
	*Drug therapy*		13	49
	*Therapy intake*		13	40
	*Adaptation to therapy*		8	11
	*Treatment costs*		5	9
	*Drug reactions*		5	7
	*Drug supply*		4	16
	*Drug allergies*		3	15
	*Central catheter management*		3	10
	*Drug box*		3	4
	*Vaccination*		2	3
	*Differences between generic medication and originator*		1	7
“I have them all, I don’t miss any (*AN, the diseases*)”. (P11) “They just wanted to cut off my leg. I resisted”. (P4) “Good thing the thrombi stopped in my ankles. The doctor told me to consider myself lucky. I could have died any day now. Thrombosis… you don’t notice it”. (P5)		**Diseases**	**21**	**66**
	*Disease effects*		10	15
	*Disease complications*		7	14
	*Other diseases*		7	11
	*Worsening disease*		5	12
	*COVID-19*		4	8
“I do it my own way. I do my own therapy scheme”. (P2) “I don’t think much about it. I know I have to take them. It is not written in any paper. I have all the boxes. I remember by looking at the boxes”. (P5) “I thought medications would be the reason for my recovery. They help”. (P5)		**Patient**	**18**	**183**
	*Patient reactions*		16	129
	*Strategies*		10	29
	*Therapy effects and recommendations*		6	13
	*Balancing drug therapy and life*		4	7
	*Habits*		3	5
“I got diabetes and did not want it”. (P2) “When she has check-ups, I have to be there to accompany her (*AN*, *the caregiver*). We go with the ambulance or the Red Cross car”. (P21) “I started taking medications, the ones I am currently taking, since I had the COVID”. (P6)		**Patient journey**	**17**	**90**
	*Check-ups*		12	27
	*Long ago*		12	21
	*Traumatic event*		6	7
	*Don’t know why it happened to me*		4	14
	*Hospitalization*		4	8
	*Details at disease onset*		3	3
	*Beginning*		2	5
	*Changing the care pathway*		2	3
	*Patient companionship*		2	2
“(*AN, At the pharmacy)* They give me medications even without prescription”. (P5) “I will remember that neurologist for the rest of my life, he destroyed my life. I was no longer eating, I had lost so much weight, I was in a pitiful state”. (P16) “The neurologist tells me that the medication I am currently taking is a lot, but that taking the medication off after all this time could trigger reactions. So he leaves the therapy as it is without changing anything”. (P13)		**Professionals**	**16**	**77**
	*Physician*		16	43
	*Hospital network*		6	12
	*Healthcare professionals*		4	12
	*Pharmacist*		3	10
“I never go on holiday (*AN, the patient’s niece and caregiver*)”. (P21) “I had a business to run. Getting sick was a luxury, I had to be as unwell as possible”. (P22)		**Family and caregivers**	**13**	**73**
	*Caregiver role*		12	63
	*Extended family*		4	6
	*Work*		2	4
“The leaflets are written too much and too small”. (P5) “Nobody told me (*AN, about drug interactions*). I even took all the medical records to the doctor. She kept them for a week but didn’t tell me what I couldn’t eat”. (P6)		**Drug information**	**5**	**6**
	*Knowing the medication leaflet*		2	2
	*Medication leaflet*		1	1
	*Complex medication leaflet*		1	1
	*Unawareness of drug interaction*		1	1
	*Adverse information*		1	1

Abbreviations: P, patient; COVID-19, coronavirus disease 19; AN, author’s note; Pt., number of patients; Oc., occurrences.

**Table 4:** PIPs in older adults for paroxetine (a randomly selected example drug).

Paroxetine	
Beers criteria 2019	
Confirmed PIP	It has a high anticholinergic effect and may cause orthostatic hypotension.
Confirmed PIP	It may exacerbate or cause hyponatremia.
PIP conditioned by a history of falls or fractures	It may cause ataxia, psychomotor impairment, syncope and falls and should be avoided in patients with a history of falls or fractures.
STOPP criteria version 2	
PIP conditioned by serum Na^+^ < 130 mmol/L	Risk of worsening symptoms in patients with hyponatriemia.

Abbreviations: PIP, potentially inappropriate prescription; STOPP, screening tool of older persons’ prescriptions.

**Table 5:** DDIs detected in the prescriptions of the study population classified by pharmacological class.

First Interacting Drug Class	Second Interacting Drug Class	Number of DDIs
NSAID	Diuretic	11
Antiplatelet agent	Antidiabetic	5
Antiplatelet agent	Diuretic	5
Antiplatelet agent	NSAID	5
Anxiolytic	Antiepileptic	4
Diuretic	ACE inhibitor	4
NSAID	Anticoagulant	3
Antidepressant	Analgesic	2
Antihistaminic	Analgesic	2
NSAID	Cardiac glycoside	2
Diuretic	ARB	2
NSAID	Corticosteroid	2
NSAID	NSAID	2
Analgesic	Analgesic	1
Antidiabetic	Antidiabetic	1
ACE inhibitor	Antigout	1
Antiplatelet agent	Analgesic	1
Antiplatelet agent	Antidepressant	1
Antiplatelet agent	Antiplatelet agent	1
Anxiolytic	Anxiolytic	1
Bronchodilator	Beta-blocker	1
Bronchodilator	Diuretic + ARB	1
Antiplatelet agent	Calcium channel blocker	1
Diuretic	Thyroid hormone	1
Analgesic	Opioid antagonist	1

Abbreviations: DDIs, drug-drug interactions; NSAID, non-steroidal anti-inflammatory drug; ACE, angiotensin-converting enzyme; ARB, angiotensin receptor blocker.

**Table 6:** Drugs with possible (score = 1) or certain (score = 2–3) anticholinergic effects in the prescriptions of the study population.

Active Ingredient	ACB Score	Patients on the Drug, n
Furosemide	1	7
Alprazolam	1	2
Atenolol	1	2
Codeine	1	2
Metoprolol	1	2
Carbamazepine	2	1
Cetirizine	2	1
Chlorthalidone	1	1
Digoxin	1	1
Fenobarbital	1	1
Loperamide	1	1
Paroxetine	3	1
Prednisone	1	1
Trazodone	1	1
Warfarin	1	1

Abbreviations: ACB, anticholinergic cognitive burden.

**Table 7:** Suggestions from researchers with expertise in drug use on how to optimise the pharmacological therapies of enrolled patients. The number of patients, along with the specific suggestion and the number of suggestions for each category, coincide.

Suggestion	Patients with the Suggestion
Deprescription of the PPI (unfavourable risk–benefit ratio)	8
Substitution of a medication with a safer alternative	8
Therapy reassessment due to the risk of adverse events or of worsening of another patient condition	6
Deprescription of a medication (lack of therapeutic indication)	3
Reduction in the number of drugs (≥5 different active ingredients) to treat a single patient condition to reduce the risk of poor medication adherence	2
New prescription of a medication to treat an untreated patient condition	2

Abbreviations: PPI, proton pump inhibitor.

## Data Availability

All data relevant to this study are included in the manuscript.

## Supplementary Materials

The following supporting information can be downloaded at: https://www.mdpi.com/article/10.3390/healthcare12020231/s1, Figure S1. Model of STS in Italian language; Table S1. PIPs detected in the study population.
