# Supplementary material for: Identification of Barriers for Active Disease Management and of Medication-Related Problems through Therapeutic Patient Education in Older Home-Care Patients
Source: Healthcare (Basel). 2024 Jan 17;12(2):231. doi: 10.3390/healthcare12020231 (PMC10815688; doi:10.3390/healthcare12020231)
Supplement: Supplementary file 1 [file healthcare-12-00231-s001.zip › healthcare-2765877-supplementary.pdf]

Supplementary Materials

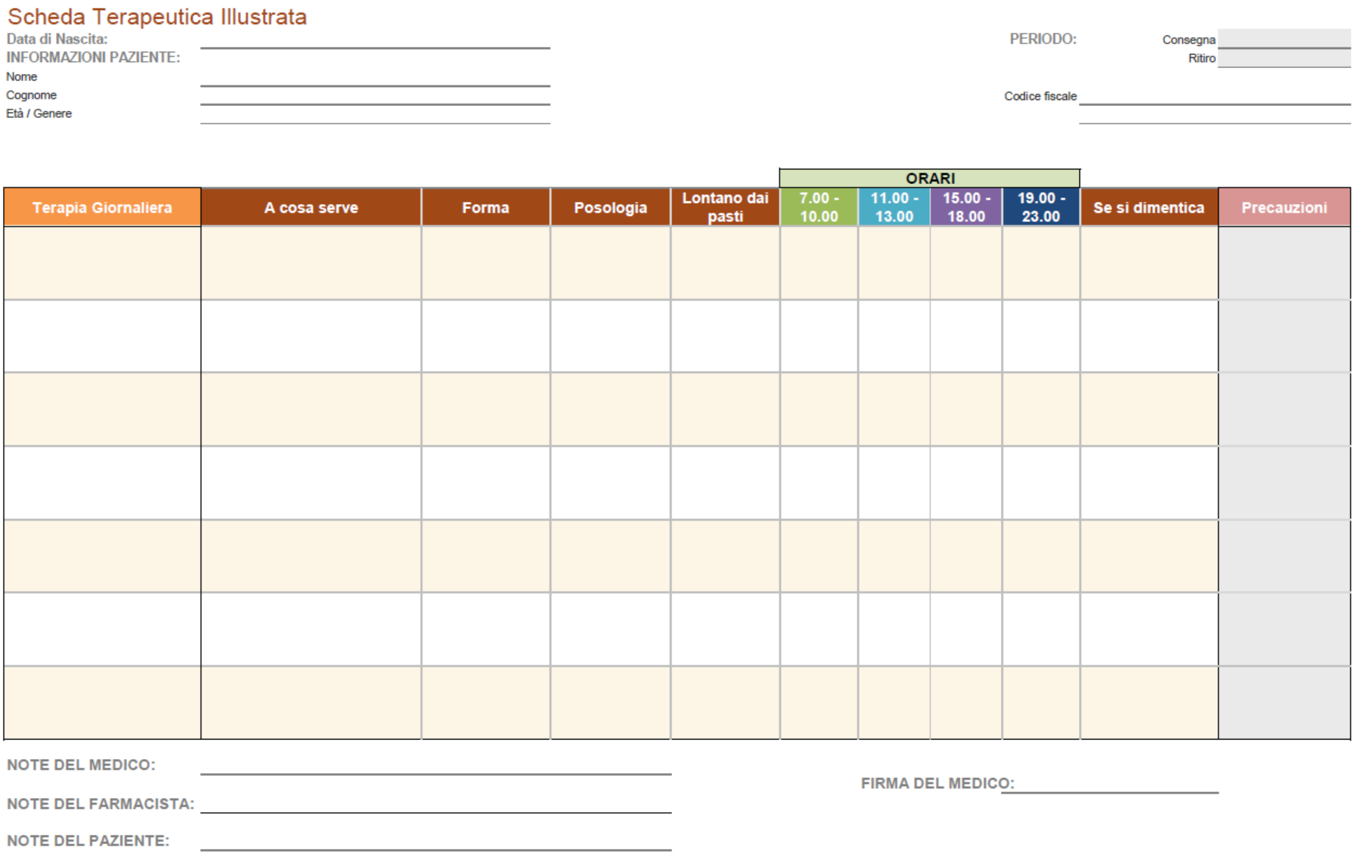

Scheda Terapeutica Illustrata

Data di Nascita:

INFORMAZIONI PAZIENTE:

Nome

Cognome

Età / Genere

PERIODO: Consegna Ritiro

Codice fiscale

Terapia Giornaliera	A cosa serve	Forma	Posologia	Lontano dai pasti	ORARI 7.00 - 10.00	11.00 - 13.00	15.00 - 18.00	19.00 - 23.00	Se si dimentica	Precauzioni

NOTE DEL MEDICO:

NOTE DEL FARMACISTA:

NOTE DEL PAZIENTE:

FIRMA DEL MEDICO:

Figure S1. Model of STS in Italian language.

Table S1. PIPs detected in the study population.

	Total	Males	Females
PIPs according to the Beers criteria, range	0-11	0-3	0-11
Confirmed PIPs	0-11	0-2	0-11
Conditional PIPs	0-3	0-2	0-3
PIPs according to the STOPP criteria, range	0-9	1-5	0-9
Confirmed PIPs	0-7	0-3	0-7
Conditional PIPs	0-4	1-3	0-4
DDIs, range	0-15	0-11	0-15
Contraindicated	0-0	0-0	0-0
Major	0-15	0-11	0-15

Abbreviations: PIPs, potentially inappropriate prescriptions.
